# Candidate biomarkers in the cervical vaginal fluid for the (self-)diagnosis of cervical precancer

**DOI:** 10.1007/s00404-017-4587-2

**Published:** 2017-11-15

**Authors:** Xaveer Van Ostade, Martin Dom, Wiebren Tjalma, Geert Van Raemdonck

**Affiliations:** 1 Laboratory of Protein Science, Proteomics and Epigenetic Signaling (PPES), University of Antwerp, Wilrijk, Belgium; 2 Centre for Proteomics (CfP), University of Antwerp, Wilrijk, Belgium; 3 Gynecological Oncology Unit, Department of Obstetrics and Gynecology, Multidisciplinary Breast Clinic, Antwerp University Hospital, University of Antwerp, Edegem, Belgium

**Keywords:** Biomarker, Cervical vaginal fluid, Cervical cancer

## Abstract

**Purpose:**

Despite improvement in vaccines against human papilloma virus (HPV), the causative agent of cervical cancer, screening women for cervical precancer will remain indispensable in the coming 30–40 years. A simple test that could be performed at home or at a doctor’s practice and that informs the woman whether she is at risk would significantly help make a broader group of patients who aware that they need medical treatment. Cervical vaginal fluid (CVF) is a body fluid that is very well suited for such a test.

**Methods:**

Narrative review of cervical (pre)cancer candidate biomarkers from cervicovaginal fluid, is based on a detailed review of the literature. We will also discuss the possibilities that these biomarkers create for the development of a self-test or point-of-care test for cervical (pre)cancer.

**Results:**

Several DNA, DNA methylation, miRNA, and protein biomarkers were identified in the cervical vaginal fluid; however, not all of these biomarkers are suited for development of a simple diagnostic assay.

**Conclusions:**

Proteins, especially alpha-actinin-4, are most suited for development of a simple assay for cervical (pre)cancer. Accuracy of the test could further be improved by combination of several proteins or by combination with a new type of biomarker, e.g., originating from the cervicovaginal microbiome or metabolome.

## Cervical cancer and the need for a bedside assay

### Current diagnosis of cervical cancer: the need for triage

Considering cervical cancer is the fourth most common female cancer worldwide, it remains a significant global problem [1], indicating that better screening and adequate interventions are necessary to reduce mortality. On the other hand, HPV vaccines offer the potential to significantly reduce the incidence of infection with an oncogenic high-risk (hr) HPV type, the causative agent for cervical cancer. Currently, two HPV vaccines are commercially available, Cervarix (GlaxoSmithKline) and Gardasil (Merck & Co). Cervarix is (cross)reactive against HPV types-16, -18, -31, -33, -45, -51 and -56, which are the seven most common cancer-causing types [2]. Gardasil is effective against HPV-16, 18 and 31, and this vaccine is also effective against HPV-6 and -11, which cause genital warts and respiratory papillomatosis [2]. A nonavalent vaccine (Gardasil 9) was recently approved that protects against nine different types of HPV (types 6, 11, 16, 18, 31, 33, 45, 52 and 58) [3–5].

However, despite the advancements in HPV vaccination, these vaccines do not cover all hr-HPV types [6] and are less efficient in women who were previously infected with HPV [7]. Together with the lack of HPV vaccination programs in many low-resource countries, these observations indicate the lasting need for population-wide cervical cancer screening programs, which further requires accurate diagnosis of the disease. Moreover, cautiously monitoring [8] vaccination programs also demands accurate detection methods.

The gradual development of cervical cancer (years) with the occurrence of several precancerous stages and the relative ease with which the tumor can be accessed offer the opportunity to screen population-wide for cervical cancer during prevention campaigns. Cervical cancer screening programs are generally based on the detection of hr-HPV via DNA or RNA assays or on the detection of cytological and/or molecular changes in cervical cells via (immuno)staining methods, such as the Papanicolaou (Pap) smear [8].

High-risk HPV DNA-based PCR tests are currently of high interest because they are 40% more sensitive at detecting a cervical abnormality than cytology [9, 10]. However, despite their higher sensitivity, these assays cannot distinguish between clinically relevant HPV infections. Indeed, approximately 80% of hr-HPV infected women spontaneously clear the virus within 1 year after acquisition [11], resulting in significant overtreatment of hr-HPV-infected women.

Cytology-based screening has several other limitations, such as the high intra- and interobserver variability, limited sensitivity, high costs and limited screening coverage [12–14]. Currently, cytology/HR-HPV DNA co-testing remains the best strategy for detecting high-grade cervical vaginal lesions [15]. In the case of positive test results, the patient is usually referred to the clinic for a colposcopy examination that detects changes in the glycogen metabolism in cervical (pre)cancerous cells [16].

The conclusion is that because of the limitations of each of these methods, screening programs are never 100% safe (false negatives). Moreover, they are subject to oversampling (high number of false positives), leading to the treatment of women with clinically irrelevant hr-HPV infections, which increases the costs and possible harm caused by the treatment. Additional triage tests, on a molecular basis, are thus necessary to provide an objective and reproducible basis for the selection of patients with clinically significant disease. Today, the best alternative is dual staining for p16^INK4a^/Ki-67 [17–19], but this method may have an increased cost and previous work has demonstrated that p16 may not have sufficient discriminatory power because normal cells also express p16 (albeit at lower levels) [20]. Therefore, alternative methods, such as the combination stainings of TOP2A and Ki-67 [21] or p16^INK4a^/Ki-67 and L1 capsid protein [22], are being sought. Unfortunately, biomarkers with good predictive values (e.g., predicting progress towards cervical carcinoma while appearing at the CIN2 stage, which still allows for treatment) do not exist yet (reviewed by [23–25]). Moreover, it is expected that a panel of biomarkers will be necessary for an accurate test that distinguishes between the several CIN states with good clinical sensitivity and specificity. Preferentially, such combined biomarkers are unrelated, e.g., molecules from different cervical cancer pathways, cancer- vs. immune-related molecules, proteins vs. (methylated) nucleic acids, such that the assay is based on a broad series of independent recognition points.

### CVF as a candidate body fluid for cervical cancer screening by self-testing

#### Self-sampling

In 2014, Arbyn et al. [26] showed that hr-HPV DNA testing on a self-sample is a way to include women who normally do not participate in regular cytology screening programs. Indeed, self-sampling has proven effective in increasing participation and screening coverage of the target population [27–32]. Many studies performed in different ethnic populations have demonstrated that self-sampling of cervical tissue via brushes, tampons, swabs or lavages is a good sample collection method for subsequent DNA genotyping, cytology or immunohistochemistry [26, 33–38]. The sampled tissue is usually resuspended in liquid buffer [39–42], although dry storage is also considered, e.g., by capping the brush [43] or by swiping the sample on paper that was chemically treated with reagents to lyse cells upon application so that they are non-infectious for safe and easy transport [44–47]. The samples are then sent to the laboratory for further analysis.

Using the emerging proteomics technologies that have become increasingly sensitive, our group and others groups have conducted several studies on the identification of the cervical vaginal proteome [48–65]. Functional classification of the CVF proteome demonstrates a significant diversity of biological roles, of which “protein metabolism and modification” and “immunity and defense” are the largest GO categories (17 and 10%, respectively). Moreover, classification based on cellular localization shows that most proteins are present in the cytoplasm or in the extracellular region (21 and 20%, respectively) [57].

Because of the immediate contact of the precancerous or cancerous tissue with the CVF, we expect that the concentrations of important cervical cancer biomarkers will be high in CVF. Unlike plasma, CVF does not contact many other tissues, and its volume is limited (milliliters versus liters). Moreover, the liquid can easily be collected by self-sampling in a non-invasive way using devices for lavages [36, 66], or using tampons [38, 67] (self-sampling devices developed before 2014 were reviewed in Othman et al. [68]). Therefore, self-sampling of CVF could overcome the practical (e.g., busy schedule, transport, and distance), emotional (e.g., fear of pain and embarrassment), and cognitive (e.g., low perceived risk and absence of symptoms) barriers that some women experience in attending cervical cancer screening programs [69–71]. Ideally, the same, self-collected sample should be used for the detection of several biomarkers, demanding minimal effort from women.

#### Self-testing

Especially in low-resource countries and remote rural areas where mail and transport are much less frequent, the continuous running of an efficient screening program for cervical cancer may demand organizational, financial and logistical efforts [72, 73] that may not always be available. A simple test, such as a lateral flow assay (LFA) that could be performed by the woman herself could be a solution to this problem because it does not require specialized instruments or personnel, and it could be performed at home or e.g., at a doctor's practice (point-of-care). LFA assays are frequently used to detect a variety of clinical analytes in plasma, serum, urine, cells, tissues and other biological samples and are also used for veterinary and industrial purposes [74–76]. Although efforts have been made to develop LFA tests for detecting HPV DNA from precancerous tissue [77], detection of proteins would be most suitable. Indeed, because of the frequent spontaneous elimination of the lesion, the presence of HPV virus does not always correspond with the presence of (pre)cancerous tissue. On the other hand, detection of proteins from precancerous cervical tissue in the cervical vaginal fluid would directly indicate the presence of such tissue. If such biomarkers could distinguish between the three CIN stages, a manual could inform the patient about whether to see a doctor. To avoid inclusion of a cell lysis step, which would compromise ease of handling, detection of secreted and/or released proteins from the precancerous lesions into the CVF is recommended for LFA.

A typical example of an LFA test that is already on the marked, is the self-test for HIV (HIVST). According to the WHO such a test used and interpreted by a self-tester can perform as well as an HIV RDT used and interpreted by a trained health worker [78] although concerns remain about test sensitivity (particularly in early infection), and linkages to care for confirmatory testing after a reactive HIVST [79]. Nevertheless, HIVST is likely to become more widely available, including in low- and medium-income countries, as it is generally accepted among key populations [80] and, therefore, has the potential to drastically increase HIV testing coverage. It is, therefore, quite possible that the HIVST LFA assay is a trendsetter in human self-diagnostic medicine.

In summary, we believe that CVF is a rich source of information regarding the physiological status of the female genital organs, including the healthy or cancerous state of the cervical region. Components from the CVF could, therefore, be used as the basis for a simple self-test/point-of-care test that, when sufficiently accurate, may overcome current problems with coverage and specificity.

## CVF biomarkers for cervical cancer

### HPV DNA assay

Many studies report on the efficiency of HPV DNA testing from self-collected cervical tissue samples, compared to samples collected by a practitioner. In most studies, HPV testing on self- and clinician-sampled specimens is similarly accurate with respect to CIN2+ detection as reported in a large cohort study [42, 81] or a meta-analysis [33], although this may depend on the test used [26].

As for detection of HPV DNA from cervical vaginal fluid there was a high agreement between the (self-sampled) CVF and the reference smears (between 89 and 93%, depending on the test used) [82] and no difference in viral load was observed when samples were collected in the estrogen-dominated proliferative phase or the progesterone-dominated secretory phase [83].

Unfortunately, HPV DNA detection requires very specialized equipment (in the previous study, a Roche cobas 4800 system); therefore, it is not suited for a self-test. Furthermore, as mentioned above, the test cannot distinguish between productive and progressive infections, resulting in a low specificity.

### Host and viral DNA methylation

With the discovery that global DNA hypomethylation progressively increases in cervical dysplasia and carcinoma [84], Widschwendter et al. [85] investigated DNA methylation in cervical vaginal specimens collected on a tampon of 11 host genes known to be methylated in cervical cancer (*SOCS1*, *CDH1*, *TIMP3*, *GSTP1*, *DAPK*, *hTERT*, *CDH13*, *HSPA2*, *MLH1*, *RASSF1A*, and *SOCS2)* and reported a correlation of the methylation status with the severity of the cervical lesion, such that invasive cervical cancers could be predicted. Along the same line, Sun and coworkers [86] analyzed in cervical vaginal lavages methylation at 14 CpG sites within the HPV16 L1 region and noticed a significant increase in methylation in samples from women with CIN3+ compared to the HPV16 genomes from women without CIN3+, indicating that hyper/hypomethylation of viral CpG sites may constitute a potential biomarker for precancerous and cancerous cervix disease [86]. In a high-throughput experiment using the Illumina 450 k DNA methylation array, Doufekis et al. [87] investigated the DNA methylation in vaginal fluid samples at more than 480,000 CpG sites and found a DNA methylation signature for cervical and endometrial cancer which resulted in a ROC area under the curve between 0.75 and 0.83.

### DNA methylation of miRNA

MicroRNAs have not only been detected in the serum or plasma of patients who are precancerous for cervical cancer (cervical adenocarcinoma and squamous cell cancer) [88], they have also been detected in CVF. In a large, randomized study of self-sampled cervical vaginal fluid, Verhoef et al. [89, 90] investigated direct DNA methylation of miR-124-2 and MAL genes on samples that tested positive for HPV and showed that DNA methylation analysis is non-inferior to cytology triage in the detection of CIN2 or higher. 2 years later, the combination of miR-124-2 methylation and methylation of another gene, FAM19A4, was investigated in a large cohort of HPV positive women by the same group [91]. The accuracy of the assay was similar for CVF self-collected samples and for clinician-collected cervical smears.

### Exosomes

Interestingly, after silencing HPV E6/E7 oncogene expression in HPV-positive cervical cells, such as HeLa cells, a distinct seven-miRNA-signature was identified in the exosomes secreted by the HeLa cells, which was accompanied by significant downregulation of let-7d-5p, miR-20a-5p, miR-378a-3p, miR-423-3p, miR-7-5p, miR-92a-3p and upregulation of miR-21-5p [92]. Later, similar results were obtained in keratinocytes transduced with E6 and E7 from mucosal HPV-16 or cutaneous HPV-38 [93]. This raised the idea of using CVF-derived exosomes for diagnostic purposes. Indeed, Liu et al. [94] described microRNA-21 and microRNA-146a to be upregulated in cervical cancer patients in association with the high levels of cervical cancer-derived exosomes in CVF, and Zhang et al. [95] recently showed that expression of the HOTAIR, MALAT1 and MEG3 long noncoding RNAs (lncRNAs) was predominantly observed in cervical cancer-derived exosomes in cervical vaginal lavage samples.

Hence, it is clear that DNA methylation or RNAs could serve as a CVF biomarker for intraepithelial cancerous lesions; however, analogous to DNA PCR, a methylation-specific or RNA-specific PCR reaction requires skilled people and specialized instruments, making it unsuitable for self-testing.

### First discovered protein markers

The discovery that carcinoembryonic antigen (CEA), CA19-9 and CA125 were present in the CVF of patients with cervical cancer or with a cervical precancerous lesion led to optimism in the 80 s that these biomarkers could help in detecting cervical cancer or its precancerous stages [96–99]. Later, it was found that these antigens were normal constituents of vaginal fluid and that their distribution was not only affected by cancer of the genital tract but also by pregnancy [100, 101] and inflammation [102], limiting their applicability for use as biomarkers. Nevertheless, the presence of CA125 in the CVF has also been correlated with endometrial cancer [103].

### Immunological proteins

In a later study [104, 105], in 60% of the patients with HPV 16 positive cervical cancer, anti-HPV 16 E7 specific IgG antibodies were found in cervicovaginal washings and sera, while no IgG reactivity was found in healthy individuals. Moreover, IgG antibody reactivity in cervicovaginal washings was higher than in the paired serum samples. Nevertheless, because the presence of these antibodies was less clear in premalignant tissue and since they could only be detected in 60% of the patients, the sensitivity and specificity are not sufficient for biomarker purposes. The same group analyzed the presence of various cytokines in cervicovaginal washings of healthy volunteers, CIN patients and cervical cancer patients and demonstrated alterations in the local cervical immune environment in cervical cancer patients. Indeed, the IL-12p40, IL-10, TGF-beta1, TNF-alpha and IL-1beta levels were significantly higher in patients with cervical cancer than in controls and CIN patients [106, 107]. Although these results are of interest for the development of immune modulating therapies and vaccination strategies, they cannot be used for diagnostic applications because no differences were seen between CIN patients, and the cytokine levels may vary according to other infections.

Since then, many studies were undertaken to identify cervical (pre)cancer protein biomarkers from swab samples or biopsies [23–25, 108, 109], yet no other cervical cancer biomarker proteins were found in the CVF.

### Alpha-actinin-4

#### Discovery

In a differential proteomics study on CVF samples from six healthy and six precancerous (CIN I, II and III) women, we identified 16 candidate biomarkers ([63], Table 1). From these, alpha-actinin-4 (ACTN4) was absent and present in all samples from healthy and precancerous women (*p* = 0.001), respectively. ELISA on 28 additional samples showed a discriminatory potential of ACTN4 at 18 pg/ml protein extract between samples from healthy and both low-risk and high-risk HPV-infected women (*p* = 0.009). Analyzing the ACTN4 concentration in 26 CVF samples originating from longitudinal studies on 9 women who experienced an HPV infection, who had a persistent infection or who cleared the virus showed that the ACTN4 levels correlated with increasing, persisting or decreasing presence of HPV E6 DNA [63].

**Table 1:** Samples used for calculating the ACTN4 discriminatory power as a biomarker for cervical (pre)cancer

Cohort	Sample	Group	Condition	Genotype	Viral load (copies/cell)	Colposcopy	Cytology	Collection medium	ACTN4 (/mg prot)
Van Raemdonck et al. [63, 64]	H07		Healthy	HPV neg		Normal	Normal	5% AA	42.8
	H12		Healthy	HPV neg		Normal	Normal	5% AA	0.6
	H52		Healthy	HPV neg		Normal	Normal	5% AA	6.1
	H54		Healthy	HPV neg		Normal	Normal	5% AA	1.9
	H62		Healthy	HPV neg		Normal	Normal	5% AA	7.2
	H20		Healthy	HPV neg		Normal	Normal	5% AA	0.5
	H05		Healthy	HPV neg		Normal	Normal	5% AA	3.1
	H64		Healthy	HPV neg		Normal	Normal	5% AA	3.6
	H28		Healthy	HPV neg		Normal	Normal	5% AA	0.0
	H69		Healthy	HPV neg		Normal	Normal	5% AA	5.3
	H70		Healthy	HPV neg		Normal	Normal	5% AA	0.1
	H08		Healthy	HPV neg		Normal	Normal	5% AA	8.7
	H14		Healthy	HPV neg		Normal	Normal	5% AA	7.5
	H73		Healthy	HPV neg		Normal	Normal	5% AA	3.5
	H87		Healthy	HPV neg		Normal	Normal	5% AA	0.0
	H90		Healthy	HPV neg		Normal	Normal	5% AA	8.2
	H09		Low risk	6	96,249	ASCUS	Normal	5% AA	12.7
	H35		Low risk	11	51,740	ASCUS	CIN1	5% AA	10.7
	H182		Low risk	6	1.00	Normal	Normal	5% AA	20.7
	H213		Low risk	6	0.05	Normal	Normal	5% AA	29.9
	P24		High risk	16/39	7729/1661	ASCUS	CIN3	5% AA	17.1
	P27		High risk	52	129	LSIL	CIN1	5% AA	113.9
	P60		High risk	16/31/52/66	0.02/11/12/31	LSIL	CIN1	5% AA	32.6
	P61		High risk	16/31/39/52/66	288/0.20/4/7416/179	LSIL	CIN1	5% AA	22.2
	P41		High risk	16/58	11571/253	HSIL	CIN2	5% AA	45.0
	P36		High risk	16/53/58/59	9126/79/2733/1510	LSIL	CIN1	5% AA	70.4
	P70		High risk	35	5159	LSIL	CIN2	5% AA	15.3
	P40		High risk	31	1696	HSIL	CIN1	5% AA	15.5
Additional samples (cohort Van Raemdonck et al. [63])	205		Healthy	HPV neg		Normal		5% AA	0.0
	207		Healthy	HPV neg		Normal		5% AA	0.0
	211		Healthy	HPV neg		Normal		5% AA	0.0
	212		Healthy	HPV neg		Normal		5% AA	0.0
	229		Healthy	HPV neg		Normal		5% AA	36.1
	206		High risk	HPV neg		ASCUS		5% AA	154.5
	204		High risk	45		LSIL/HSIL		5% AA	0.0
	210		High risk	16		LSIL		5% AA	52.1
	224		High risk	18/39/56		LSIL		5% AA	0.0
	225		High risk	56		LSIL		5% AA	0.0
Van Raemdonck et al. [63, 64] (longitudinal samples)	42	Patient L1	High risk new infection	52/53/59/66	178/0.07/50/6	LSIL		5% AA	0.5
	119			52	26.00	Normal		5% AA	5.0
	308			16/52	171/283	ASCUS		5% AA	22.7
	85	Patient L2	High risk clearing	33/52/58/66	0.01/9147/268/526	LSIL		5% AA	32.1
	146			HPV neg	0.00	LSIL		5% AA	11.2
	281			HPV neg	0.00	Normal		5% AA	0.5
	36	Patient L4	High risk persisting	16/53/58/59	9126/79/2733/1510	LSIL		5% AA	17.7
	105			16/53/58	99,999/11/4123	LSIL		5% AA	37.1
	172			16/58	3/99,999	LSIL		5% AA	39.7
	290			58	4997.00	LSIL		5% AA	46.0
	154	Patient L5	Healthy	HPV neg	0.00	Normal		5% AA	4.2
	242			31	0.62	Normal		5% AA	0.5
	302			HPV neg	0.00	Normal		5% AA	2.0
	15	Patient L6	High risk clearing	16/39/53	2627/12,052/0.51	LSIL		5% AA	10.1
	135			16	4.00	Normal		5% AA	0.5
	271			16	33.00	Normal		5% AA	1.8
	S266	Patient L7	High risk clearing	16/31/51/56	111/0.2452/33/271	LSIL		5% AA	15.0
	23			HPV neg	0.00	Normal		5% AA	0.5
	127			HPV neg	0.00	Normal		5% AA	3.1
	359			HPV neg	0.00	Normal		5% AA	1.3
	43	Patient L8	High risk new infection	HPV neg	0.00	Normal		5% AA	0.5
	147			51/59	99,999/46	LSIL		5% AA	17.4
	348			51/59	0.13/165	ASCUS		5% AA	7.2
	70	Patient L9	High risk clearing	35	5159.00	HSIL		5% AA	6.3
	177			HPV neg	0.00	Normal		5% AA	0.6
	342			HPV neg	0.00	Normal		5% AA	0.5
	103	Patient L10	High risk persisting	31	351.00	Normal		5% AA	18.1
	218			31	1556.00	Normal		5% AA	25.0
	343			31	3479.00	ASCUS		5% AA	60.8
Berlin cohort	DS77		High risk	16/31/52			CIN3	PBS	0.0
	DS78		High risk	16			CIN3	PBS	896.2
	DS72		Cancerous	16			CxCa	PBS	782.9
	DS73		Cancerous	HPV neg			CxCa after conisation	PBS	719.0
	DS80		Cancerous	18/56			Cx AdenoCa	PBS	355.2
	DS86		Cancerous	16			CxCa	PBS	463.8
	DS90		Cancerous	HPV neg			CxCa FIGO IIIb N1 (1/10)	PBS	559.0
	DS74		Cancerous	16			CxCa 1a1 VAIN III	PBS	2316.7
	DS79		Cancerous	16			ZxCa susp. Peritoneal	PBS	3075.2
	DS84		Cancerous	HPV neg			CxCa	PBS	500.0
	DS87		Cancerous	HPV neg			CxCa pT1a2 G2 L1 V0 R1	PBS	178.1
	DS88		Cancerous	16			CxCa FIGO IIa	PBS	949.2
	DS89		Cancerous	16			CxCa FIGO IIb	PBS	1354.8
	DS91		Cancerous	18/43			CxCa FIGO lib	PBS	636.4
Van Raemdonck et al. [64]	5714	No HIV	ESN population	HPV neg		–	–	PBS	2.5
	3896	No HIV	ESN population	HPV neg		–	–	PBS	4.2
	6624	HIV	ESN population	HPV neg		–	–	PBS	0.0
	6589	HIV	ESN population	HPV neg		–	–	PBS	0.0
	6488	HIV	ESN population	HPV neg		–	–	PBS	0.0

Three different cohorts were included with a varying CVF sample size and collection medium. A series of samples from the first cohort consisted of 28 singular samples and samples taken at different time points from 9 patients (longitudinal samples) [63]. These were further supplemented with ten additional singular samples from the same cohort. Fourteen samples were from another cohort (Charité, Berlin; Charite IRB Ethics Approval EA02/129/08), consisting of samples from two women with CIN III and twelve women with different stages of cervical cancer. We also included five CVF samples from a previously described cohort [64]. From this cohort, all samples were HPV-negative, as demonstrated by RT-PCR genotyping [161], and three of them came from HIV-positive women. Classification was made based on colposcopy examination and/or cytology results. In case both examinations gave conflicting results, colposcopy results had priority. Samples from healthy individuals were given a gray background. Since the study by Van Raemdonck et al. [64] lacked colposcopy and cytology, the absence of (pre)cancerous tissue was decided on the basis of HPV absence

#### Alpha-actinin-4 and cancer

Alpha-actinin-4 is predominantly expressed in cellular filopodia and lamellipodia, and as such, it is important for the formation of cell protrusions and migration [110]. Experiments in colon and pancreatic cancer cells have shown that ACTN4 overexpressing cells are highly mobile and have a significantly increased metastatic ability [111–114]. Apart from in colorectal and pancreatic cancer, the protein is also overexpressed in ovarian cancer, osteosarcoma, lung cancer, oral squamous cell carcinoma, salivary gland carcinoma, bladder cancer, breast cancer and esophageal cancer (for an overview, see [115]). ACTN4 gene amplifications were shown to correlate with pancreatic cancer [113] and could be a potential biomarker for metastatic potency and for predicting the effectiveness of chemoradiotherapy in locally advanced pancreatic cancer [116]. Elevated levels of ACTN4 contribute to the increased migratory potential of neuroblastomas [117]. Worsened survival rates were seen in ACTN4 overexpressing ovarian tumors [118]. Moreover, studies have demonstrated that in addition to its role in cytoskeleton remodeling, ACTN4 interacts with signaling mediators, chromatin remodeling and transcription factors. Nuclear localization of the protein was seen in different tumors [110, 119, 120], and recruitment of ACTN4 to the *pS2* promotor, an estrogen receptor (ER) target in the ER-positive breast cancer cell line MCF7, suggested that ACTN4 may play a role in E2-mediated regulation of breast cancer proliferation [121, 122]. Interestingly, ACTN4 has been reported to be present in exosomes from tumor (mesothelioma) cells [123]. ACTN4 thus functions as a promoter for many tumor types and could be an important target protein in drug development. Therefore, it is not surprising to see the protein appearing in the cervical vaginal fluid of women who have cervical precancerous lesions. ACTN4 may, therefore, play an important role in the development of a simple bedside assay for cervical cancer based on CVF components.

#### Efficiency of alpha-actinin-4 as a CVF biomarker for cervical cancer

**Fig. 1:**
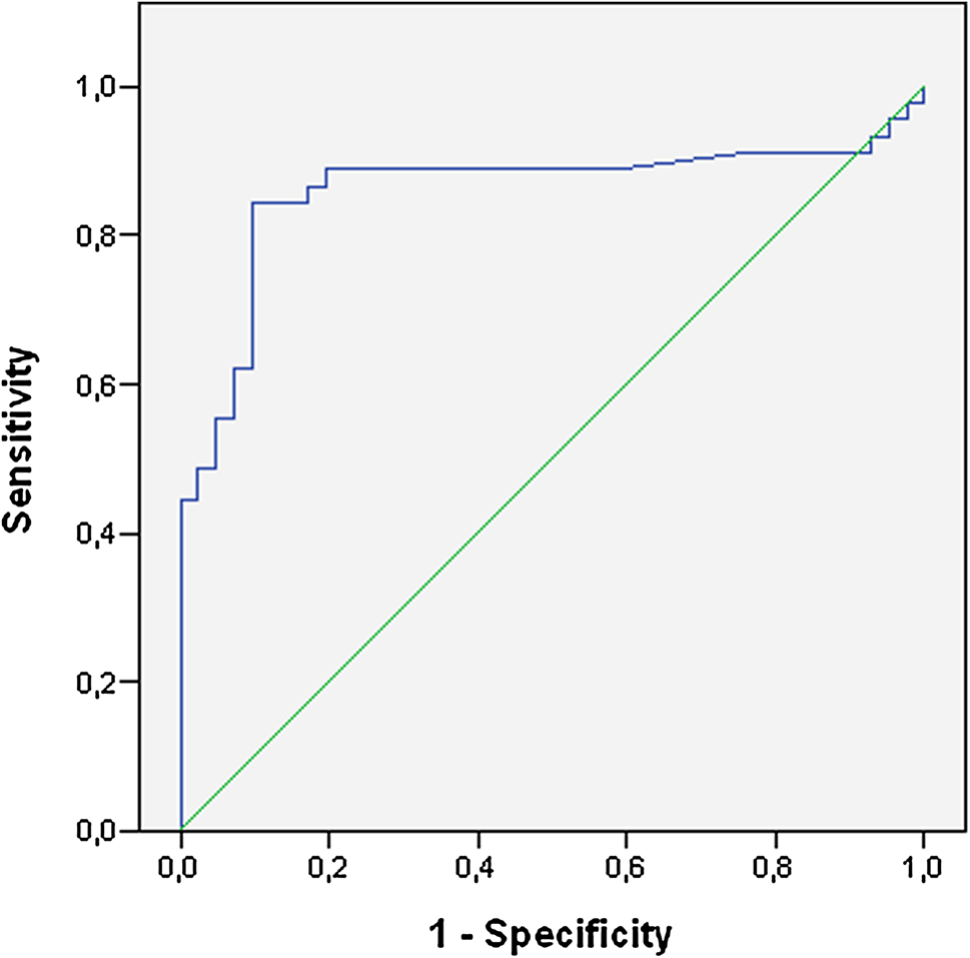
ACTN4 ROC curve for discrimination between the healthy and anomalous (ASCUS, CIN I and higher) state. Data from Table 1 were used. Numbers of samples for healthy and anomalous state were 48 and 43, respectively. The ROC curve was created by SPSS with inclusion of cutoff values for positive classification. Sensitivity and specificity values were, respectively, 84 and 86% when a cutoff value of 10 pg ACTN4/mg total protein was used, resulting in an area under the curve (AUC) of 86%

For a preliminary determination of the sensitivity and specificity of ACTN4 as a cervical (pre)cancer biomarker, we extended our sample pool with 10 CVF samples from the previous cohort [63], 14 CIN III or cervical cancer samples from a Berlin cohort (see Table 1) and five samples from an African cohort, three of which were from women infected with HIV-1 [64] (Table 1). Based on colposcopic determination of the precancerous state, we divided the samples into two classes. The first class (*N* = 43) contained samples originating from healthy women, while the second class (*N* = 43) contained samples originating from women with small (ASCUS, LSIL) or larger (HSIL) signs of precancerous tissue or with cancerous tissue. Because the samples came from different hospitals in different volumes, we took the total mass of protein as a reference instead of the sample volume. For this normalization, the ACTN4 concentration was recalculated as pg/mg total protein instead of pg/ml. The resulting ROC curve showed an AUC of 86% (Fig. 1) with a sensitivity (true positives/true positives + false negatives) and specificity (true negatives/true negatives + false positives) of 84 and 86%, respectively, when a cutoff value of 10 pg ACTN4/mg total protein was used. It must be mentioned that this value was obtained despite differences in the volumes and collection media resulting from the inclusion of different cohorts. Because only a limited number of samples from women with precancerous tissue above CIN II or CIN III were included in this study (*N* = 6), it was not possible to correlate the ACTN4 concentration with different CIN stages. However, clear overexpression of ACTN4 was visible in all cancerous samples. Interestingly, none of the three samples from HIV-1 infected (HPV negative) individuals scored above the cutoff value, suggesting that HIV-1 infection does not interfere with the ACTN4 levels in CVF. Studies in our lab are currently ongoing to examine the correlation of the ACTN4 concentration with the CIN stages and to evaluate the CVF concentration in women infected with additional sexually transmitted viruses, bacteria and protozoa.

### Network biomarkers

**Fig. 2:**
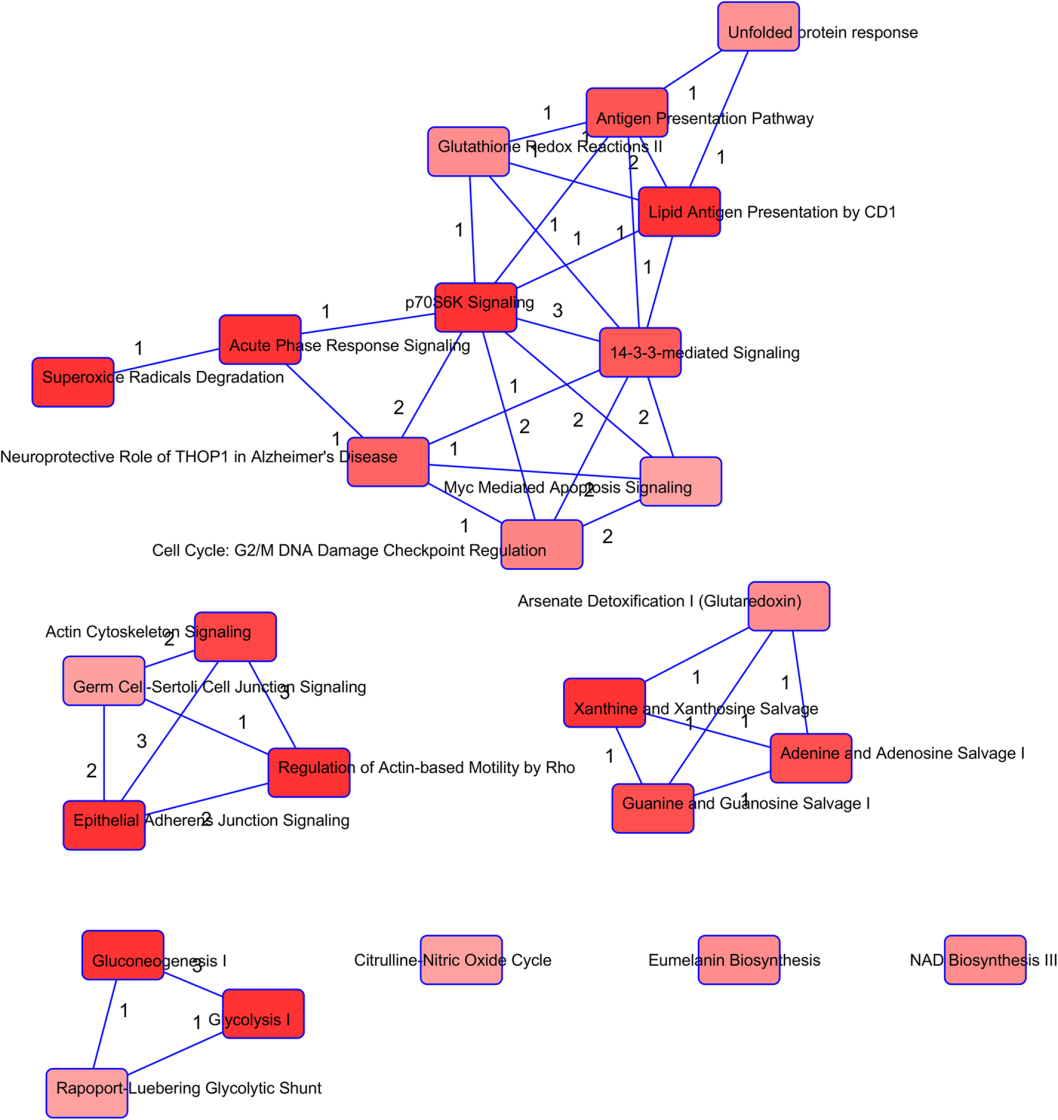
Overlap of canonical pathways containing proteins from Table 2 after IPA Core Analysis. The degree of grayness defines the *p* value, where deeper red stands for the lowest* p* values. All *p* values are < 0.05. The numbers accompanying edges represent common proteins within the 2 connected canonical pathways

Assuming that precancerous tissue is (partially) attacked by the immune system, we hypothesized that ACTN4 is released in the CVF from lysed epithelial cells with many other intracellular factors, including those involved in the development of precancerous lesions and/or cervical cancer. Aberrant concentrations of (some of) these factors in the CVF may indicate there is growing precancerous tissue and, therefore, an increased chance of developing a malignant tumor. Therefore, starting from the protein lists we obtained from the differential proteomics study on CVF from healthy and precancerous patients [63], protein IDs (Table 2) were introduced into the Ingenuity Pathway Analysis (IPA) program, and common pathways were searched [124]. Interestingly, proteins in CVF from precancerous women interconnected much more inside pathways that make up the ‘hallmarks of cancer’ described by Hanahan and Weinberg [125–127] compared to CVF proteins from healthy people. In addition, a literature search showed that CVF proteins classified by IPA in the ‘cancer’ category were more correlated with cervical cancer when they originated from the CVF of precancerous women. Moreover, many of these proteins clustered in a network with angiotensin II as a central mediator [124], and further IPA studies showed their overrepresentation in the following four clusters of pathways that belong to a cancer hallmark: (1) gluconeogenesis/glycolysis, (2) adenine/guanine metabolism, (3) adherens/tight junction formation and 4) a larger set of interconnected pathways clustered around the p70^S6K^ pathway (Fig. 2). The first two clusters are a result of increased metabolism, a typical feature of tumor cells. Interestingly, the p70^S6K^ pathway has been described to be involved in cell motility [128]; hence, the two last pathway clusters influence the migration of cells and concomitant metastatic activity. Indeed, an altered expression of tight and adherens junction proteins was frequently reported in cervical neoplasia [129–131], and Claudin-1, a component of tight junction strands, was recently described as having similar diagnostic potential as p16^INK4a^ in histological and cytological biomarker assays for cervical cancer detection [132]. Additionally, the association of ACTN4 with adherens junction formation has been described [110, 133]. Such ‘network biomarkers’, rather than single biomarkers, could increase the accuracy and prognostic value of cervical cancer diagnosis and allow us to better identify the early presence of the tumor, tumor type, and development state.

**Table 2:** Proteins that differ in CVF abundance between healthy individuals and individuals with cervical precancerous tissue (CIN I or higher)

Name	Acc. No	ID
Increased levels in CVF from women with adenocarcinoma:		
Fujii et al. [98], Harlozinska et al. [97] and McDicken et al. [96]		
Carcino embryonic antigen (CEA)	Q13984	Q13984_HUMAN
carbohydrate antigen disialyl Lewis a, CA19-9	Q969X2	SIA7F_HUMAN
Increased levels in CVF from women with precancerous lesions with stringent selection (*p* < 0.05)		
Van Raemdonck et al. [63]		
14-3-3 protein epsilon	P62258	1433E_HUMAN
Actin-related protein 3	P61158	ARP3_HUMAN
Alpha-actinin-4	O43707	ACTN4_HUMAN
Annexin A2	P07355	ANXA2_HUMAN
ATP synthase subunit beta, mitochondrial	P06576	ATPB_HUMAN
Cellular retinoic acid-binding protein 2	P29373	RABP2_HUMAN
Nicotinamide phosphoribosyltransferase	P43490	NAMPT_HUMAN
Phosphoglycerate kinase 1	P00558	PGK1_HUMAN
Putative elongation factor 1-alpha-like 3	Q5VTE0	EF1A3_HUMAN
Pyruvate kinase isozymes M1/M2	P14618	KPYM_HUMAN
Serpin B13	Q9UIV8	SPB13_HUMAN
Squamous cell carcinoma antigen 1 (SCCA-1); Serpin B3	P29508	SPB3_HUMAN
Exclusive occurrence in CVF from women with precancerous lesions and described to be involved in cervical cancer		
Van Raemdonck et al. [63] and Van Ostade et al. [125]		
14-3-3 protein theta	P27348	1433T_HUMAN
Angiotensinogen	P01019	ANGT_HUMAN
Annexin A4	P09525	ANXA4_HUMAN
Cathepsin B	P07858	CATB_HUMAN
CD59 glycoprotein	P13987	CD59_HUMAN
Ceruloplasmin	P00450	CERU_HUMAN
Gelsolin	P06396	GELS_HUMAN
High mobility group protein B2	P26583	HMGB2_HUMAN
Interleukin-18	Q14116	IL18_HUMAN
Macrophage migration inhibitory factor	P14174	MIF_HUMAN
Macrophage-capping protein	P40121	CAPG_HUMAN
Mucin-5B	Q9HC84	MUC5B_HUMAN
Myosin light polypeptide 6	P60660	MYL6_HUMAN
Phosphoglycerate mutase 1	P18669	PGAM1_HUMAN
Protein disulfide isomerase A3	P30101	PDIA3_HUMAN
Protein S100-P	P25815	S100P_HUMAN
Serpin B13	Q9UIV8	SPB13_HUMAN
Superoxide dismutase [Mn]	P04179	SODM_HUMAN

From the list of proteins identified in Van Raemdonck et al. [63], the following two subsets were distinguished: (1) proteins that were, to a high extent (*p* < 0.05), qualitatively or quantitatively different in the samples from precancerous women compared to the samples from healthy women and (2) proteins that, to a lower extent, qualitatively differed from the samples in precancerous women (presence in at least one of the six ‘precancerous’ samples, while not present in the ‘healthy’ samples), which were described to be interconnected and to play a role in cervical cancer [124]

## Perspectives

### Microbiome

Over recent decades, a substantial amount of work has been done on the characterization of the vaginal microbiome in relation to several diseases. Two groups [134, 135] described the beneficial role of *Lactobacillus* species in vaginal and reproductive health, while overgrowth of *G. vaginalis*, *A. vaginae*, *Eggerthella*, *Prevotella*, *BVAB2* and *Megasphaera type 1* as well as a marked depletion of *Lactobacillus* were essential for the diagnosis of BV [136]. In a review by Datcu et al. [137], it was shown that subgroups of bacterial vaginosis (BV) could be identified wherein single or paired bacteria were dominant.

Several studies have also pointed to a correlation of microbiota with HPV and cervical (pre)cancer. Compared to HPV-negative women, the vaginal bacterial diversity of HPV-positive women is more complex  [138], and because of an association between the cervical microbiota and CIN stages, the combined effect of the microbiota and HPV on the risk of CIN could be determined [139]. Moreover, Mitra et al. [140] showed an association of increasing CIN stage with increasing vaginal microbiota diversity, suggesting a role for microbiota in regulating viral persistence and disease progression. Such changes may be reflected in the expression levels of microbial enzymes, considering Dasari and coworkers [141] demonstrated that the microbial enzymes mucinase, sialidase, and protease were significantly (*p* < 0.01) elevated in patients with cervical dysplasia and, therefore, may serve as risk-factors for the development of cervical cancer.

Interestingly, shotgun microbiota sequencing also showed that the HPV community in healthy woman is much more complex than previously defined, suggesting that co-existing non-oncogenic HPV viruses may stimulate or inhibit the oncogenic virus via viral interference or immune cross-reaction [142].

### Metabolome

Although not yet applied for diagnosing HPV or cervical (pre)cancerous lesions, metabolomics data were recently correlated with microbiome data in BV. Alterations in amino acid, carbohydrate, and lipid metabolism were associated with the presence and concentration of specific BV bacteria [143]. Additionally, a dramatic loss of lactic acid and higher concentrations of mixed short chain fatty acids (SCFAs), including acetate, propionate, butyrate, and succinate, characterized BV [144], and Nelson and coworkers [145] reported on the importance of biogenic amines for dysbiosis and the outgrowth of BV-associated vaginal bacteria. If assays can be developed for the simple and rapid quantification of such microbiome and metabolome alterations, their combination with classical ELISA-like protein/peptide tests may lead to a simple yet very powerful diagnostic tool for detecting cervical cancer and its several precancerous stages.

### Urine

Since CVF is washed away with the first flow of urine, it is expected that especially first-void urine contains most of the CVF components, including the mucus and debris from vaginal and cervical exfoliated cells. This may explain why the first collected part of a urine void collected with a special device (Colli-Pee™, Novosanis, Belgium) contains more human and HPV DNA than the subsequent parts [146, 147]. Moreover, self-sampling of urine for subsequent HPV DNA tests was very well accepted by patients [148, 149] and provided sensitivity for CIN2+ detection comparable to a physician-taken smear or brush-based self-sample [150]. Several groups have attempted to identify urine components, other than HPV DNA, that could distinguish between healthy and precancerous states. The nature of these components varies from the hormone ratio [151] and collagen abundance [152] to host and/or viral gene methylation [153, 154]. However, although some of these studies show encouraging results, further validation is recommended with standardized protocols and higher patient numbers. Nevertheless, this does not exclude that biomarkers identified from experiments with CVF could be evaluated in urine and vice versa.

### Alternative techniques

Although their complexity still prevents the development of self-tests or medical practice applications, several biophysical applications are currently being evaluated for high-throughput testing of CVF samples. Fourier transformed infrared spectroscopy (IR) was performed on 25 cervical vaginal lavage specimens from women referred for colposcopy [155]. For the CIN III stage, the authors showed a strong correlation between IR spectra and histopathology; however, less precise matching was seen for lower CIN grades. Therefore, it is possible that the differences seen in IR spectroscopy reflect the molecular abnormalities in cervical cells during progression to cancer. If so, the technique may help in clinical decision making, but more studies are required to make this a routine technique in cervical cancer screening.

Additionally, mass spectrometry could contribute to cervical cancer diagnosis. The technology could very well be used in specialized laboratories where samples are collected, and it could complement or replace current methods, such as cytology and immunohistochemistry. However, at present, the main limitation of MS techniques lies in the sensitivity. For instance, to detect alpha-actinin-4, we used a targeted LC–MS technique called multiple reaction monitoring (MRM), but so far we have been unsuccessful because the limit of detection of the LC-Triple Quadrupole system for ACTN4 in CVF was at least ten-fold higher than the cutoff value of 18 pg/ml. Whether CVF contains cervical cancer biomarker proteins that are sufficiently high in abundance for detection by mass spectrometry remains to be elucidated, but at least for CVF labor biomarkers, Brown and co-workers [156] showed that differences in proteome profiles were visible after protein separation on weak cation exchange chips and analysis using Surface-Enhanced Laser Desorption Ionization Time-of-Flight Mass Spectrometry (SELDI-TOF–MS). Differences were attributed to fragments of alpha- or beta-hemoglobin. A fragment of alpha-hemoglobin was found to potentiate smooth muscle cell contraction in response to bradykinin, oxytocin and prostaglandin-F2alpha. Recently, Cricca et al. [157] compared a commercial kit for HPV genotyping with a Matrix-Assisted Laser Desorption Ionization-Time Of Flight (MALDI-TOF) method, developed to genotype 16 high-risk human papillomavirus (HPV) types in cervical cytology specimens, and concluded that the MALDI-based method is well-suited for broad spectrum HPV genotyping in large-scale epidemiological studies. A very elegant integration of cytology/histology methods and molecular tests could come from MALDI-imaging whereby the mass spectrum is recorded from a thin tissue section, allowing for localization of different analytes to become visible. In this way, the distribution of many proteins and their expression profiles in cytological samples were correlated with the histological features and Pap groups, allowing for unbiased and automated classification of cervical Pap smears [158].

## Conclusion

In conclusion, several components residing in the cervical vaginal fluid are valuable candidate biomarkers for diagnostic tests for cervical cancer or its precancerous states. Since CVF and CVF-containing first-void urine are appropriate body fluids for use in self-tests or point-of-care tests, we could focus in the future on those biomarkers that lend itself to the development of such tests. Proteins are excellent candidates for this, and may originate from the virus, the tumor, the host immune system or the disturbed microbiome. For this, alpha-actinin-4 may offer a very good starting point, but we still have a way to go. Continued investigation is necessary to define a CVF/urine panel of biomarkers with which we can move forward to a standardized evaluation protocol such as in the PRoBE study design [159]. Moreover, when the biomarker(s) should be used for clinical decisions, impact on patient outcome must also be evaluated with great care [160].
